# Cooperation between ZEB2 and Sp1 promotes cancer cell survival and angiogenesis during metastasis through induction of survivin and VEGF

**DOI:** 10.18632/oncotarget.23139

**Published:** 2017-12-11

**Authors:** Dongjoon Ko, Semi Kim

**Affiliations:** ^1^ Immunotherapy Convergence Research Center, Korea Research Institute of Bioscience and Biotechnology, Daejon, Korea; ^2^ Department of Functional Genomics, Korea University of Science and Technology, Daejon, Korea; ^3^ Department of Chemistry, Korea Advanced Institute of Science and Technology, Daejon, Korea

**Keywords:** ZEB2, cell survival, angiogenesis, Sp1, VEGF

## Abstract

Epithelial-mesenchymal transition (EMT) is a process implicated in tumor invasion and metastasis. During EMT, epithelial cells undergo molecular changes to acquire mesenchymal phenotypes, which are mediated by EMT-inducing transcription factors. Previously, we showed that ZEB2 cooperates with the transcription factor Sp1 to function as a transcriptional activator of vimentin, integrin α5, and cadherin-11, which promotes cancer cell invasion. We hypothesized that ZEB2, through cooperation with Sp1, would mediate diverse cellular functions beyond EMT and invasion during metastasis. ZEB2 upregulated the expression of Sp1-regulated genes such as survivin, bcl-2, cyclin D1, and vascular endothelial growth factor in an Sp1-dependent manner, resulting in increased cancer cell survival and proliferation and endothelial cell activation *in vitro*, and increased circulating tumor cell survival and tumor angiogenesis *in vivo.* In addition, Sp1 enhanced ZEB2 stability, suggesting the presence of a positive feedback loop between ZEB2 and Sp1. Clinical data showed that ZEB2 expression was positively associated with Sp1 expression, and that the expression of both of these factors had prognostic significance for predicting survival in cancer patients. This study suggests that invasion is linked to cancer cell survival and angiogenesis by ZEB2 during cancer progression, and increases our understanding of the pathways via which EMT-inducing transcription factors regulate the complex process of metastasis.

## INTRODUCTION

The metastatic cascade is a multistep process consisting of several steps, including local invasion, intravasation, cancer cell survival in the circulation, extravasation, micrometastasis, and metastatic colonization [1]. As an initial step in cancer metastasis, epithelial tumor cells are activated to invade the surrounding stromal tissues through the epithelial-mesenchymal transition (EMT) process [2, 3]. EMT is a process by which cells undergo morphological and molecular changes to acquire a more mesenchymal phenotype and is a common occurrence in metastasis and cancer progression [4, 5]. During EMT, epithelial cells gradually lose their epithelial markers, such as E-cadherin, and concomitantly acquire mesenchymal markers, such as vimentin [4]. These changes are usually mediated by EMT-inducing transcription factors, including members of the Snail, ZEB, and basic helix-loop-helix families, directly or indirectly [6–8]. These transcription factors induce EMT, including E-cadherin downregulation, and cancer cell migration and invasion. In addition, these transcription factors confer cells with a self-renewal capacity, enhanced resistance to apoptosis and anoikis, an ability to override senescence, and proangiogenic and proinflammatory activities [7, 8], although the underlying mechanisms remain unclear. For example, while enforced expression of the constitutively active p65 NF-kB subunit in MCF10A cells results in upregulation of ZEB2/SIP1 and ZEB1 to induce EMT, small interfering RNA (siRNA)-mediated ZEB2 or ZEB1 depletion in MCF10A/p65 cells leads to cell death instead of the expected EMT reversal, MET, suggesting that the cells are dependent on ZEB2 and ZEB1 for survival [9]. In addition, ZEB2 protects bladder cancer and squamous carcinoma cells from cisplatin- and ultraviolet-induced apoptosis, and this pro-survival effect of ZEB2 is independent on ZEB2-mediated EMT induction and cell cycle arrest [10, 11]. Expression of EMT-inducing transcription factors such as Snail1, Snail2, and Twist1 often correlates with enhanced tumor microvessel vasculature and expression of angiogenic factors [7]. However, the underlying molecular mechanisms remain to be determined.

We have been investigating the roles of ZEB2 in the induction of mesenchymal genes during cancer cell invasion and metastasis. Previously, we reported that ZEB2 cooperates with the transcription factor Sp1 to function as a transcriptional activator of mesenchymal genes such as vimentin, integrin α5, and cadherin-11 to induce invasion [12, 13]. Therefore, we hypothesized that, by cooperating with Sp1, ZEB2 drives diverse cellular functions other than EMT and tumor invasiveness during tumor progression. In particular, we aimed to determine whether cooperation between ZEB2 and Sp1 promotes cancer cell survival and paracrine activation of endothelial cells. In this study, we found that ZEB2 upregulated survivin, cyclin D1, and vascular endothelial growth factor (VEGF) through cooperation with Sp1 to promote cancer cell survival and proliferation and endothelial cell activation. Furthermore, ZEB2 induced tumor angiogenesis and circulating tumor cell (CTC) survival *in vivo*. In addition, Sp1 modulated ZEB2 stability, indicating the presence of bidirectional regulation between ZEB2 and Sp1. Clinically, ZEB2 and Sp1 were positively associated. This study demonstrates previously unrecognized novel roles of ZEB2 protein in cancer cell survival and angiogenesis beyond invasion during cancer progression.

## RESULTS

### ZEB2 modulates expression of Sp1-regulated genes

We previously reported that ZEB2 functions as a transcriptional activator of mesenchymal genes such as vimentin, integrin α5, and cadherin-11 to induce invasion by cooperating with the transcription factor Sp1. Sp1 can modulate important genes involved in diverse cellular functions including cell survival, cell cycle progression, and angiogenesis [14, 15], and Sp1 is reported to upregulate survivin, bcl-2, cyclin D1, VEGF, and VEGFR [15, 16]. Therefore, we attempted to explore whether ZEB2 can modulate the transcription of certain factors involved in other cellular functions besides invasion.

**Figure 1:**
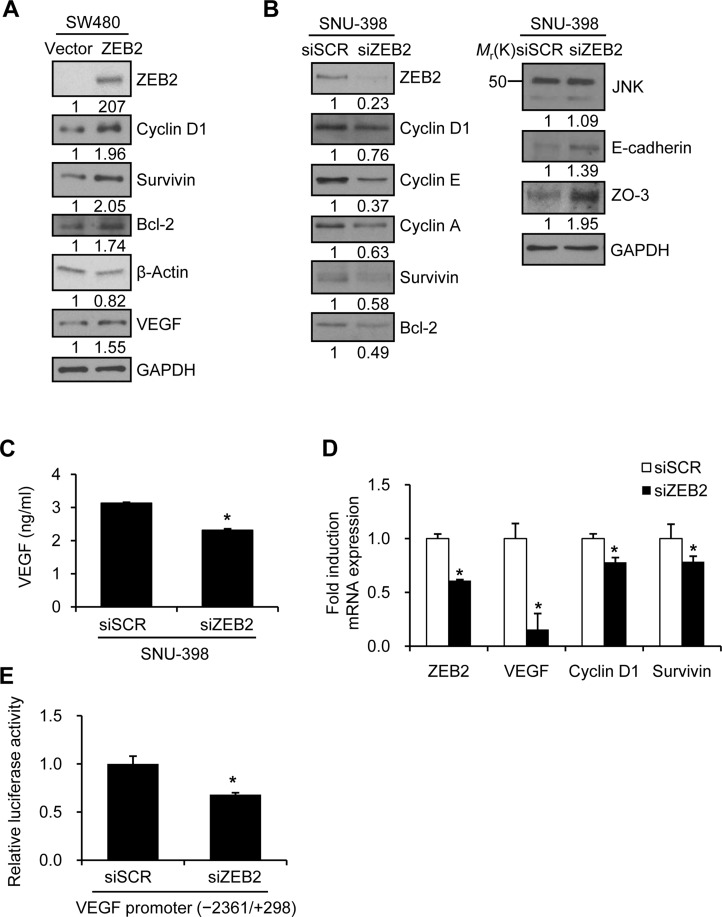
ZEB2 modulates expression of Sp1-regulated genes (**A**) SW480 cells were transfected with a ZEB2 expression vector for 48 h. Transfected cells were lysed and used for immunoblotting. An anti-myc antibody was used to detect myc-tagged ZEB2. GAPDH or β-actin was used as an internal control. (**B**–**D**) SNU-398 cells were transfected with ZEB2-specific siRNA or scrambled siRNA for 48 h. (B) Transfected cells were lysed for immunoblot analysis. Densitometry quantification was performed on the immunoblots, using GAPDH as a loading control. (C) Conditioned medium from transfected cells was collected for an additional 48 h. VEGF levels in conditioned medium were determined by an ELISA. (D) Real-time qPCR analysis of ZEB2, VEGF, cyclin D1, and survivin mRNA levels. (**E**) SNU-398 cells were co-transfected with ZEB2-specific siRNA and a VEGF promoter (−2361/+298) reporter construct. Firefly luciferase activity representing VEGF promoter activity was measured after 48 h and normalized to the fluorescence signal intensity to measure the transfection efficiency. Values represent mean standard deviation. ^*^P < 0.05. siSCR, scrambled siRNA.

Transient transfection of SW480 cells, which normally express little to no endogenous ZEB2, with a ZEB2 expression construct upregulated survivin, bcl-2, cyclin D1, and VEGF expression (Figure 1A). Suppression of ZEB2 by siRNA in SNU-398 cells, which express high levels of endogenous ZEB2, reduced expression of survivin, bcl-2, cyclin D1, cyclin E, and cyclin A, whereas expression of E-cadherin and ZO-3 increased as expected (Figure 1B). The results of an ELISA showed that VEGF secretion was significantly reduced by suppression of ZEB2 in SNU-398 cells (Figure 1C).

In addition, real-time qPCR analysis showed that VEGF was downregulated by knockdown of ZEB2 (Figure 1D) and upregulated by ZEB2 overexpression (see below). To explore whether suppression of ZEB2 reduces VEGF promoter activity, SNU-398 cells were transiently co-transfected with siRNA specific to ZEB2 and a reporter plasmid driven by the VEGF promoter (−2361/+298). Knockdown of ZEB2 significantly reduced VEGF promoter activity by 32% (Figure 1E). Survivin and cyclin D1 mRNA expression was also reduced by knockdown of ZEB2 (Figure 1D).

### ZEB2 induces transcription of VEGF, cyclin D1, and survivin in an Sp1-dependent manner

**Figure 2:**
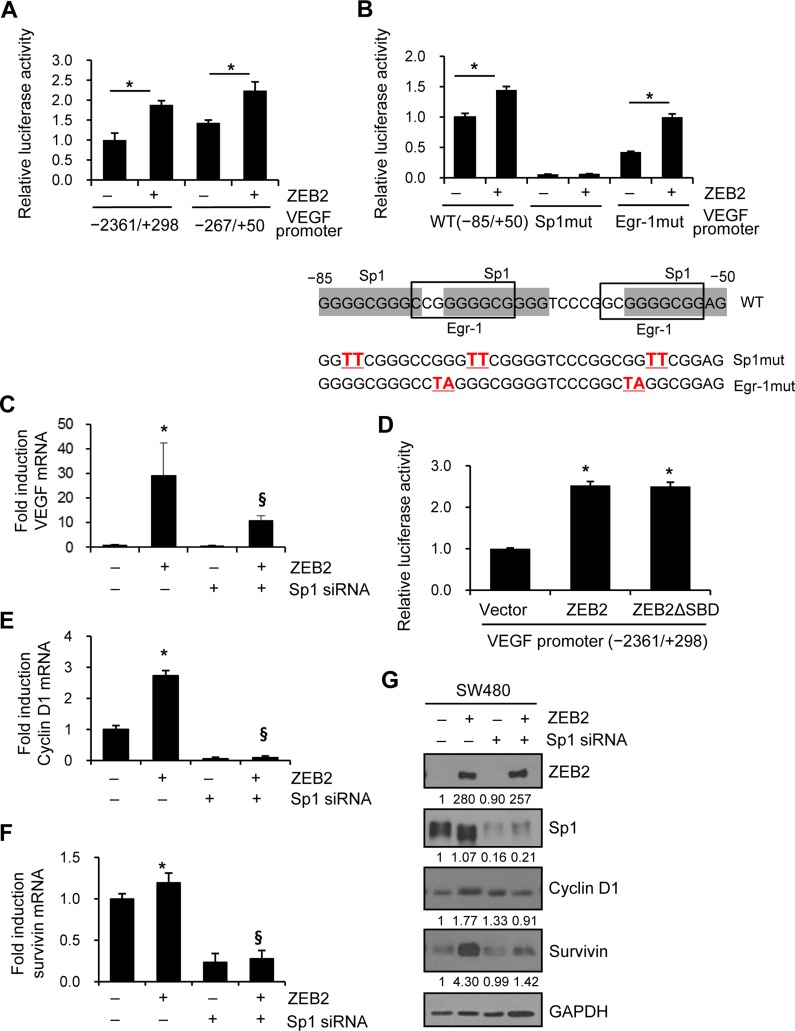
ZEB2 induces transcription of VEGF, cyclin D1, and survivin in an Sp1-dependent manner (**A**) SW480 cells were co-transfected with a ZEB2 expression vector and VEGF promoter (−2361/+298 and −267/+50) reporter constructs for 48 h. Firefly luciferase activity representing VEGF promoter activity was measured after 48 h and normalized to Renilla luciferase activity to measure the transfection efficiency. (**B**) Mutation analysis of Sp1 sites and Egr-1 sites in the VEGF promoter (−85/+50). Reporter constructs containing Sp1 site or Egr-1 site mutations were used in the reporter assay with SW480 cells. Values represent mean standard deviation. ^*^P < 0.05. (**C, E, F, G**) SW480 cells were co-transfected with the ZEB2 expression vector and Sp1-specific siRNA for 48 h. (C) Real-time qPCR analysis to determine the effect of Sp1-specific siRNA on VEGF mRNA induction by ZEB2 in SW480 cells. (D) Reporter assay to determine the effect of mutant ZEB2 lacking the Smad-binding domain on VEGF promoter activity. (E, F) Real-time qPCR analysis of the mRNA levels of cyclin D1 (E) and survivin (F) in SW480 cells. Values represent mean ± standard deviation. ^*^P < 0.05 compared with empty vector + control siRNA; ^§^*P* < 0.05 compared with ZEB2 + control siRNA. (G) Transfected cells were lysed for immunoblot analysis. An anti-myc antibody was used to detect myc-tagged ZEB2. Densitometry quantification was performed on the immunoblots, using GAPDH as a loading control.

We then explored whether Sp1 is involved in ZEB2-mediated VEGF transcription. A reporter assay showed that ZEB2 significantly upregulated VEGF promoter (−2361/+298 and −267/+50 regions) activity in SW480 (Figure 2A) and HEK293E (Supplementary Figure 1A) cells. Three Sp1-binding sites and two Egr-1-binding sites are present in the −85/−50 region and are reported to be involved in VEGF transcription [17, 18].

We analyzed the functional involvement of the Sp1 sites in the −85/−50 region by performing reporter assays using mutated VEGF promoter constructs. Mutation of the Sp1 sites resulted in a drastic decrease in ZEB2-induced activation of the VEGF promoter in SW480 (Figure 2B) and HEK293E (Supplementary Figure 1B) cells, indicating the functional significance of the proximal Sp1 sites for the effects of ZEB2. Of note, mutation of the Sp1 sites also dramatically decreased basal VEGF promoter activity, which is consistent with previous reports [17], suggesting the possible involvement of these sites in basal VEGF promoter activity. By contrast, mutation of the Egr-1 sites did not dramatically change ZEB2-induced VEGF promoter activity, although it partially reduced basal VEGF promoter activity (Figure 2B and Supplementary Figure 1B).

We also explored whether Sp1 is required for ZEB2-induced VEGF transcription. Real-time qPCR analysis showed that ZEB2-mediated transcription of VEGF was diminished in SW480 cells following knockdown of Sp1 by siRNA (Figure 2C). In addition, a reporter assay demonstrated that mutant ZEB2 lacking the Smad-binding domain (amino acid residues 437–487) activated VEGF promoter to a similar extent as wild-type ZEB2 in SW480 cells (Figure 2D), suggesting that ZEB2 upregulated VEGF expression in a Smad-independent, but Sp1-dependent, manner.

We also explored the function of Sp1 in ZEB2-mediated cyclin D1 and survivin expression. Real-time qPCR analysis showed that ZEB2-mediated transcription of cyclin D1 (Figure 2E) and survivin (Figure 2F) was reduced in SW480 cells following knockdown of Sp1 by siRNA. Immunoblot analysis showed that Sp1 was required for ZEB2-induced survivin and cyclin D1 expression (Figure 2G). Together, these results suggest that ZEB2 induces VEGF, cyclin D1, and survivin in an Sp1-dependent manner.

### ZEB2 promotes HUVEC proliferation through upregulation of VEGF

**Figure 3:**
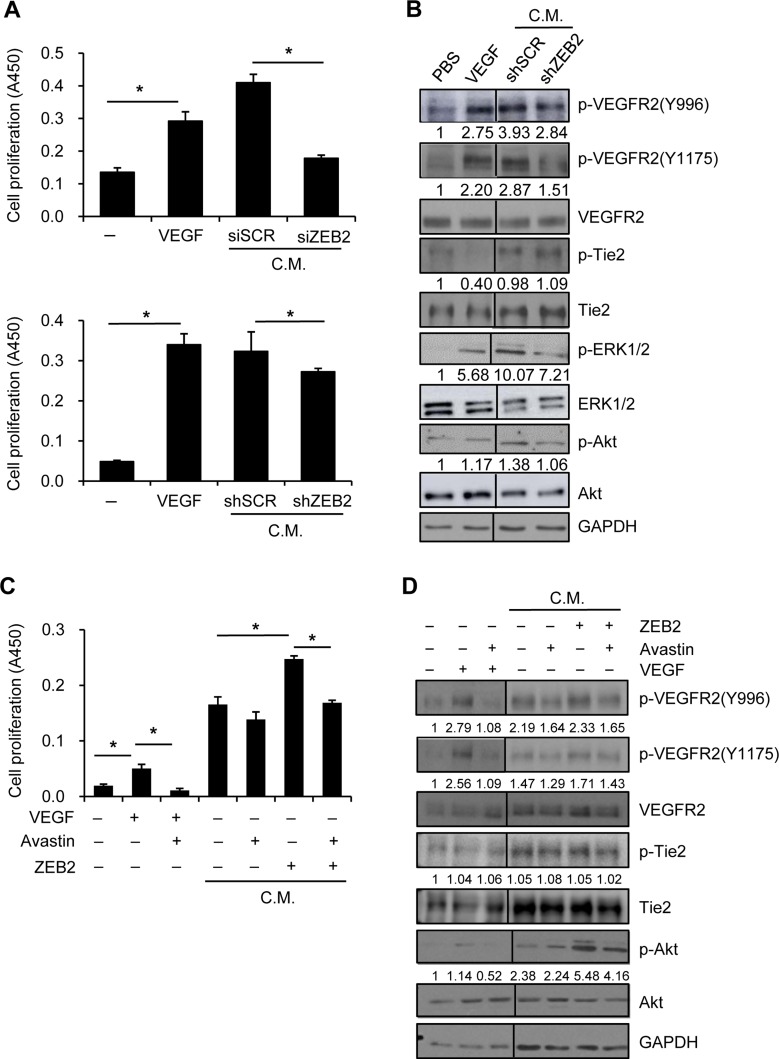
ZEB2 increases HUVEC proliferation through upregulation of VEGF (**A**) SNU-398 cells were transiently transfected with ZEB2-specific siRNA or scrambled siRNA for 48 h (Upper). Alternatively, ZEB2-suppressed SNU-398 stable cells were incubated for 48 h (Lower). Then, conditioned medium was collected for an additional 48 h. HUVECs were seeded into 96-well plates and incubated for 24 h before serum-starvation for 24 h. HUVECs were incubated with conditioned medium from transfected SNU-398 cells or with 10 ng/ml VEGF for 48 h (A) or 10 min (**B**). Cell proliferation was determined by the colorimetric WST assay (A) and cells were lysed for immunoblot analysis (B). siSCR, scrambled siRNA; shSCR, scrambled shRNA. (**C**, **D**) Serum-starved HUVECs were incubated for 48 h (C) or 10 min (D) in the presence or absence of a VEGF-blocking antibody (10 µg/ml) along with the conditioned medium from HEK293E cells transfected with the ZEB2 expression vector for 48 h. Cells treated with 10 ng/ml VEGF were included. Cell proliferation was determined by the colorimetric WST assay (C). Values represent mean standard deviation. ^*^P < 0.05. Cells were lysed for immunoblot analysis (D). C.M., conditioned medium. Densitometry quantification was performed on the immunoblots; phospho-VEGFR2, phospho-Tie2, phospho-ERK1/2, and phospho-Akt were normalized against the corresponding total protein levels.

VEGF is a well-known potent proangiogenic factor and activator of endothelial cells. To characterize VEGF activity induced by ZEB2, SNU-398 cells were transiently transfected with ZEB2-specific siRNA or scrambled siRNA. HUVECs were serum-starved and then cultured in conditioned medium derived from SNU-398 cells transfected with siRNA for 48 h. Conditioned medium from ZEB2-suppressed cells decreased HUVEC proliferation compared with conditioned medium from control cells (Figure 3A upper). In addition, SNU-398 cells were stably transfected with a shRNA plasmid targeting ZEB2 or scrambled shRNA (see below). Conditioned medium from ZEB2-suppressed stable cells also decreased HUVEC proliferation compared with conditioned medium from control stable cells (Figure 3A lower).

To examine the effects of conditioned medium from ZEB2-suppressed stable SNU-398 cells on VEGFR2 activation and subsequent intracellular signaling, serum-starved HUVECs were incubated with conditioned medium for 10 min. Phosphorylation of VEGFR2 was substantially induced by VEGF (10 ng/ml) or conditioned medium from control cells, but not by conditioned medium from ZEB2-suppressed cells (Figure 3B). On the other hand, phosphorylation of Tie2 was not apparently altered, suggesting that angiopoietins are not involved in ZEB2-mediated HUVEC activation. Autophosphorylation of VEGFR2 has been reported to activate intracellular signal mediators such as Akt, MAPK, and focal adhesion kinase in endothelial cells [19]. Phosphorylation of Akt and ERK1/2 was also induced in HUVECs by conditioned medium from control cells compared with conditioned medium from ZEB2-suppressed cells (Figure 3B).

In addition, HEK293E cells were transiently transfected with a ZEB2 expression vector. The results of an ELISA showed that VEGF secretion was significantly induced by ZEB2 overexpression (Supplementary Figure 2). Proliferation of HUVECs incubated with conditioned medium from ZEB2-overexpressing HEK293E cells was significantly higher than that of HUVECs incubated with conditioned medium from control cells. Furthermore, HUVEC proliferation was significantly reduced following the addition of a VEGF-blocking antibody to the conditioned medium from ZEB2-overexpressing cells (Figure 3C), suggesting that VEGF is a critical secreted mediator of pro-angiogenic activity induced by ZEB2. Phosphorylation of VEGFR2 and Akt induced by conditioned medium from ZEB2-overexpressing cells was reduced by a VEGF-blocking antibody (Figure 3D). Together, these results suggest that ZEB2 directly induces VEGF production and thus stimulates endothelial cell activation in a paracrine manner.

### ZEB2 promotes cancer cell survival and proliferation

**Figure 4:**
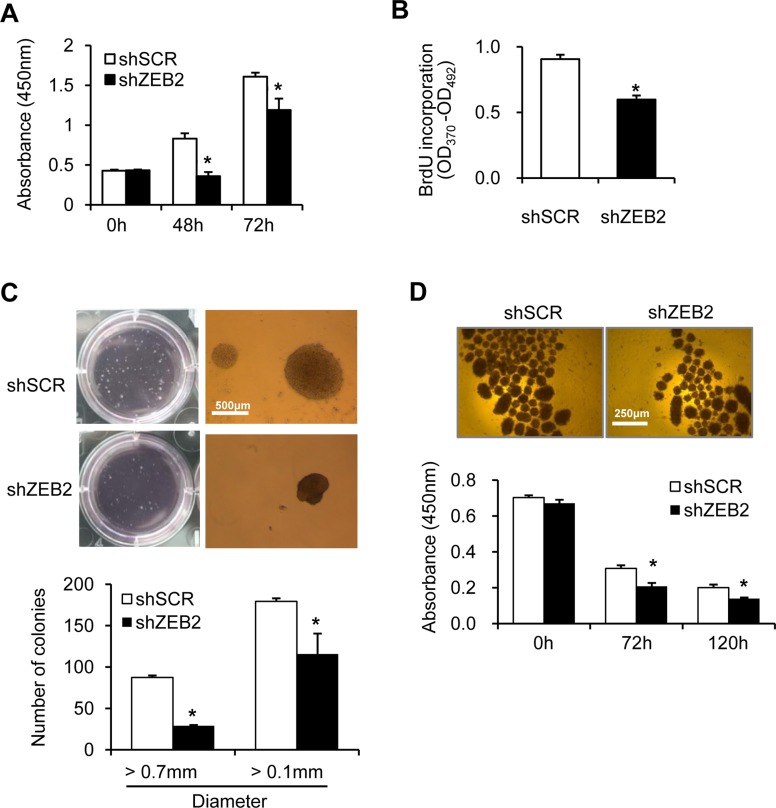

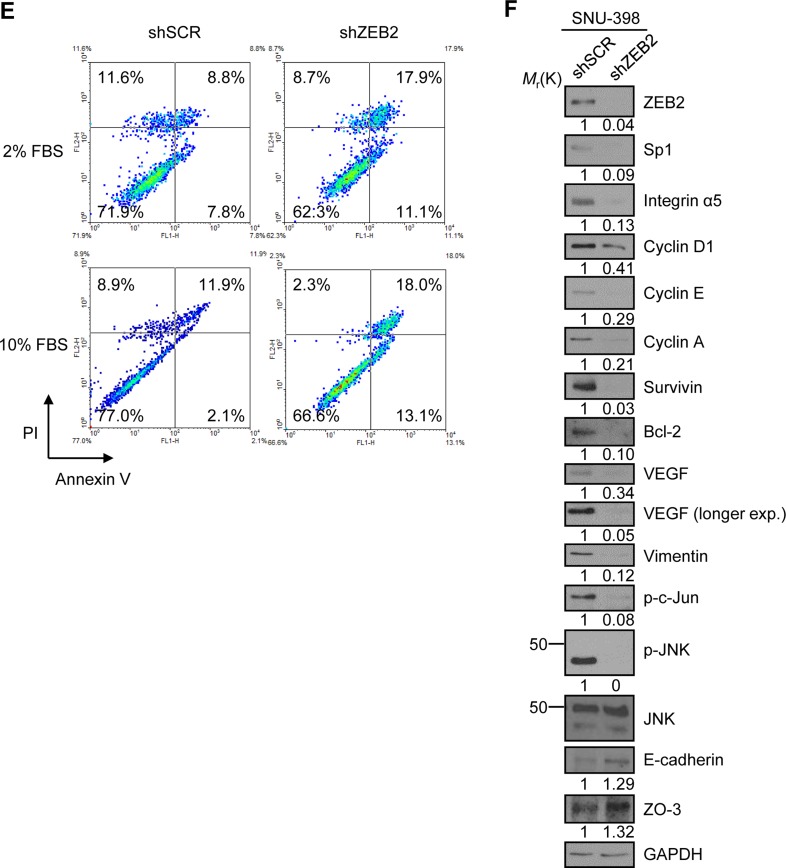
ZEB2 promotes cancer cell proliferation and survival (**A**) ZEB2-suppressed SNU-398 stable cells were seeded into 96-well plates at a density of 4 × 10^3^ cells/well and incubated for up to 72 h. Cell proliferation was determined by the colorimetric WST assay. (**B**) BrdU incorporation assay. ZEB2-suppressed SNU-398 stable cells were seeded into 96-well plates at a density of 5 × 10^3^ cells/well and incubated for 48 h. Then, the cells were labeled with 10 mM BrdU for 2 h and the assay was performed as described in the Materials and Methods. (**C**) Soft agar anchorage-independent growth assay. SNU-398 stable cells were seeded into 6-well plates at a density of 1 × 10^3^ cells/well in triplicates in semi-solid agar and then allowed to grow for 14 days. Total numbers of colonies (>0.1 mm) and colonies with a diameter of >0.7 mm were counted. Bar, 500 µm. (**D**) To induce anoikis, SNU-398 stable cells were seeded into 96-well plates with an Ultra-Low Attachment Surface at a density of 3 × 10^4^ cells/well and grown for up to 5 days. Representative images of cells at 5 days are shown. Bar, 250 µm. Cell viability was determined by the colorimetric WST assay. Values represent mean ± standard deviation. ^*^P < 0.05. (**E**) SNU-398 stable cells were incubated for 48 h with 2% or 10% FBS under suspension culture conditions and then stained with annexin V and PI for flow cytometric analysis. (**F**) SNU-398 stable cells were incubated for 48 h and then lysed for immunoblot analysis. shSCR, scrambled shRNA. Densitometry quantification was performed on the immunoblots, using GAPDH as a loading control except that phospho-JNK was normalized against total JNK protein.

To investigate the effects of ZEB2 on cancer cell proliferation and survival, SNU-398 cells were stably transfected with ZEB2-specific siRNA or scrambled siRNA. Depletion of ZEB2 was confirmed by immunoblot analysis. Knockdown of ZEB2 reduced invasion, as expected (Supplementary Figure 3). Suppression of ZEB2 significantly decreased cell proliferation over 3 days (Figure 4A). BrdU incorporation analysis revealed that depletion of ZEB2 significantly reduced S-phase progression of SNU-398 cells (Figure 4B). A soft agar assay showed that ZEB2-suppressed cells grew fewer and smaller colonies over 14 days than control cells (Figure 4C), indicating that anchorage-independent growth of cells was reduced by ZEB2 knockdown. When cells were incubated under suspension culture conditions for up to 5 days to induce anoikis, survival of ZEB2-suppressed cells was 30% lower than that of control cells (Figure 4D). Flow cytometric analysis also showed that ZEB2 suppression resulted in a moderate increase in apoptosis induction (Figure 4E), indicating that ZEB2 is able to confer anoikis resistance to cancer cells *in vitro*.

Immunoblot analysis revealed that suppression of ZEB2 substantially reduced expression of cyclin D1, cyclin E, cyclin A, survivin, bcl-2, and VEGF, whereas expression of E-cadherin and ZO-3 increased (Figure 4F). Consistent with our previous report [13], expression of Sp1, integrin α5, and vimentin was also reduced by ZEB2 suppression in SNU-398 cells (Figure 4F). We also previously reported that ZEB2 indirectly activates JNK signaling through integrin α5 and cadherin-11 in SW480 cells [13]. Similarly, phosphorylation of JNK and c-Jun was reduced by ZEB2 knockdown in SNU-398 cells, indicating that the JNK pathway is involved in ZEB2-mediated cellular functions.

We examined possible cooperation between ZEB1 and Sp1 because ZEB1, the other member of the ZEB family, also induces EMT and invasion [7]. Analysis of The Cancer Genome Atlas (TCGA)-generated colorectal adenocarcinoma data (two cancer studies; TCGA, Nature 2012 [20], and TCGA, Provisional) showed that expression of ZEB1 is lower than or comparable to that of ZEB2 (Supplementary Figure 4). HepG2 and SNU-638 cells, which express moderate to high levels of endogenous ZEB1, were transiently transfected with ZEB1-specific siRNAs. Immunoblot analysis revealed that suppression of ZEB1 did not substantially reduce expression of Sp1, survivin, cyclin D1, or VEGF in HepG2 cells, nor did it reduce expression of Sp1, VEGF, or bcl-2 in SNU-638 cells (although survivin was reduced by ZEB1 knockdown; Supplementary Figure 5). These results suggest that ZEB2, but not ZEB1, cooperates specifically with Sp1 to induce Sp1-regulated genes.

### ZEB2 promotes tumor angiogenesis and CTC survival *in vivo*

**Figure 5:**
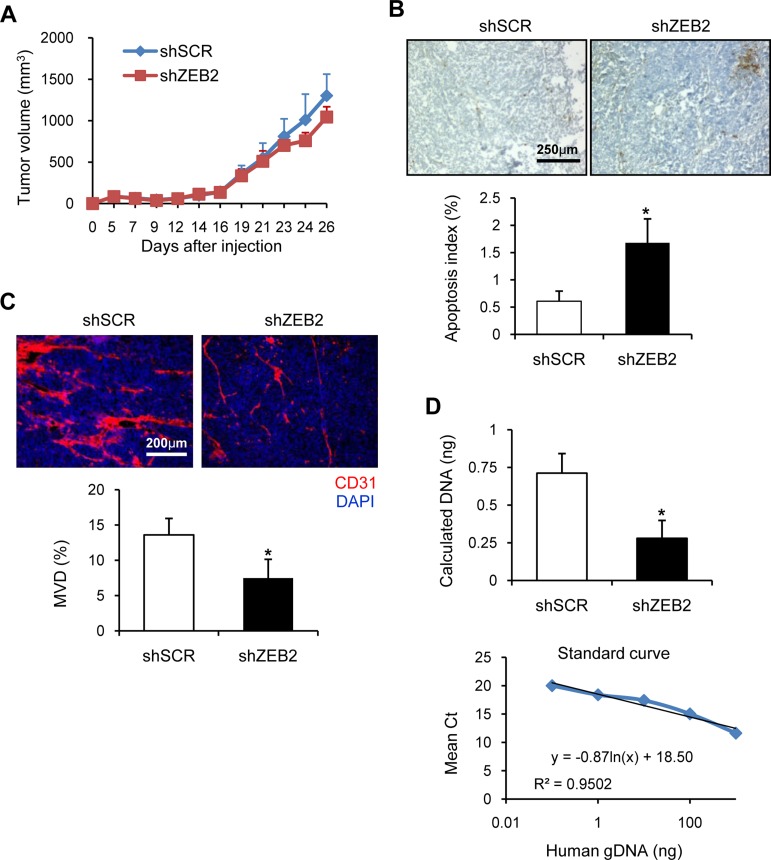
ZEB2 promotes tumor angiogenesis and CTC survival (**A**) SNU-398 stable cells (1 × 10^7^ cells/mouse) were injected subcutaneously into the flanks of nude mice (*n* = 8) as described in the Materials and Methods. Body weight (Supplementary Figure 6) and tumor volume were measured for 26 days. Tumor volume was calculated using the formula, length × width^2^ /2. (**B**) TUNEL staining of tumor sections from (A) was performed to measure the level of apoptosis. Representative images are shown. The apoptosis index (%) was determined by calculating the number of TUNEL-positive cells relative to the total number of cells, which consisted of at least 1000 cells per field. Five randomly selected fields of tumor sections per mouse were analyzed, excluding necrotic areas. Bar, 250 µm. (**C**) Tumor sections from (A) were stained to detect endothelial cells using the anti-CD31 antibody. Red, CD31-positive cells; blue, DAPI. Representative images are shown. MVD (%) was calculated based on the ratio of the CD31-positive area to the total observation area. Eight random fields in tumor sections per mouse (*n* = 8 for each group) were captured and analyzed. Bar, 200 µm. (**D**) SNU-398 stable cells (5 × 10^6^ cells/mouse) were intravenously injected into nude mice (*n* = 4). At 24 h after injection, lungs were removed to extract total DNA. Real-time qPCR analysis was performed of human PTGER2 with total DNA extracted from lungs. The amounts of human genomic DNA initially present in the qPCR reaction tube were estimated (Upper) from the standard curve produced by real-time qPCR using human total DNA extracted from SNU-398 parental cells, which was mixed with mouse total DNA from lungs of nude mice (Lower). Values represent mean ± standard deviation. ^*^P < 0.05. shSCR, scrambled shRNA.

To evaluate the effects of ZEB2 on tumor growth and angiogenesis *in vivo*, ZEB2-suppressed SNU-398 stable cells were injected subcutaneously into the flanks of nude mice. Tumors tended to grow slower in mice injected with ZEB2-suppressed cells than in those injected with scrambled shRNA-transfected cells (control cells), although this only reached borderline significance at the level of *P* = 0.06 on day 26 (Figure 5A). TUNEL staining of tumor sections showed that the level of apoptosis in tumors from mice injected with ZEB2-suppressed cells was significantly higher than that in tumors from mice injected with control cells (Figure 5B). The limited correlation between the apoptotic index and tumor volume may be due (at least in part) to the possibility that tumor growth in this model is influenced mainly by proliferative activity rather than apoptosis.

To delineate whether ZEB2 suppression influenced tumor angiogenesis, we analyzed microvessel density (MVD) in tumors from mouse xenografts. Microvessels were detected by CD31 immunostaining, and the density of CD31-positive cells in tumor sections was determined by computer-assisted quantification. MVD in tumors from mice injected with ZEB2-suppressed cells was significantly lower than in those from mice injected with control cells (Figure 5C), suggesting that ZEB2 induces tumor angiogenesis *in vivo*.

An EMT signature is prominent in CTCs in experimental and clinical samples [21], although the precise mechanisms remain unclear. From the *in vitro* results, we hypothesized that ZEB2 might play a role in survival of CTCs by directly upregulating anti-apoptotic factors such as survivin and bcl-2. Therefore, we investigated whether ZEB2 modulates survival and subsequent seeding/arrest of CTCs (early metastasis). As an early metastasis model, ZEB2-suppressed or control cells were intravenously injected into nude mice. At 24 h after injection, mice were sacrificed to immediately remove the lungs and total DNA was extracted from the lungs. Real-time qPCR analysis was performed of human PTGER2 genomic DNA using 1 µg of total DNA as a template to determine the tumor cell content in the lungs, and then the amounts of human genomic DNA initially present in the qPCR reaction tube were extrapolated from the standard curve. The amount of human genomic DNA was significantly lower in the lungs of mice injected with ZEB2-suppressed cells than in the lungs of mice injected with control cells (Figure 5D). On the other hand, human PTGER2 genomic DNA was not substantially detected in the blood of either group of mice (data not shown), suggesting that most CTCs were rapidly removed from the circulation. These results suggest that ZEB2 supports early metastasis probably by upregulating cell survival through induction of survivin and bcl-2.

### Positive feedback loop between ZEB2 and Sp1

**Figure 6:**
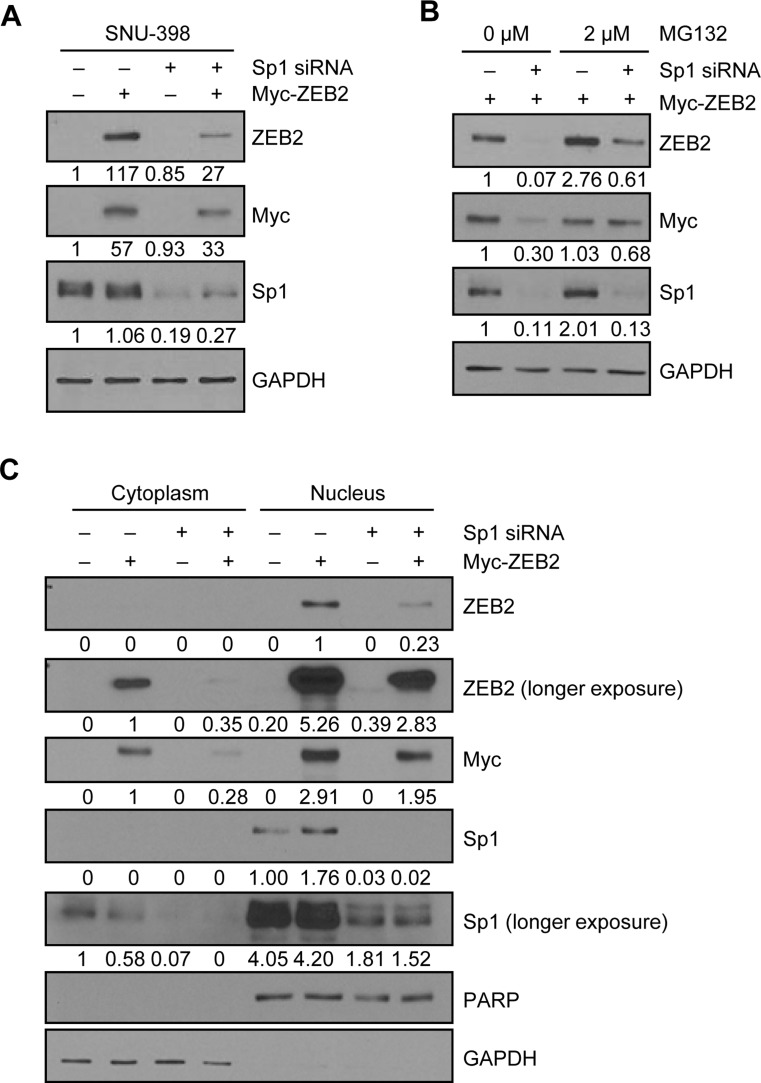
Positive feedback loop between ZEB2 and Sp1 (**A**) HEK293E cells were transiently co-transfected with the ZEB2 expression vector and Sp1-specific siRNA for 48 h prior to lysis for immunoblot analysis. (**B**) HEK293E cells co-transfected with the ZEB2 expression vector and Sp1-specific siRNA for 42 h were treated with MG132 (2 µM) for 6 h before lysis for immunoblot analysis. (**C**) A cytosolic fraction and a nuclear fraction were prepared from HEK293E cells co-transfected with the ZEB2 expression vector and Sp1-specific siRNA for 48 h for immunoblot analysis. GAPDH and PARP were used as internal controls for the cytosolic and nuclear fractions, respectively. An anti-myc and an anti-ZEB2 antibodies were used to detect myc-tagged ZEB2. Densitometry quantification was performed on the immunoblots, using GAPDH or PARP as a loading control.

To investigate the mechanisms underlying ZEB2-Sp1 cooperation, HEK293E cells were transiently co-transfected with a ZEB2 expression vector and Sp1-specific siRNA. Immunoblot analysis showed that suppression of Sp1 dramatically reduced the level of ZEB2 (Figure 6A, lane 2 vs. lane 4). Treatment with the proteasome inhibitor MG132 substantially reversed the reduction in ZEB2 by Sp1 suppression (Figure 6B), indicating that Sp1 suppression induced proteasome-dependent degradation of ZEB2 in HEK293E cells. Subcellular fractionation and immunoblot analysis confirmed that suppression of Sp1 reduced both the cytoplasmic and nuclear levels of ZEB2, although ZEB2 was mainly present in the nucleus (Figure 6C). Immunocytochemistry also showed that both the cytoplasmic and nuclear ZEB2 levels were reduced by knockdown of Sp1 (Supplementary Figure 7).

On the other hand, in ZEB2-overexpressing cells, the nuclear Sp1 level was increased and the cytoplasmic Sp1 level was decreased compared with control cells while Sp1 was mainly present in the nucleus (Figure 6C), suggesting that ZEB2 induces Sp1 nuclear transport/localization. Of note, the upregulation of Sp1 by ZEB2 overexpression is consistent with our previous result in SNU-398 cells, where ZEB2 upregulated Sp1 through binding to Sp1 and thus enhancing Sp1 protein stability [13]. Together, our previous results and the present study suggest that ZEB2 enhances Sp1 stability and induces Sp1 nuclear localization.

### Clinical significance of the ZEB2-Sp1 association

**Figure 7:**
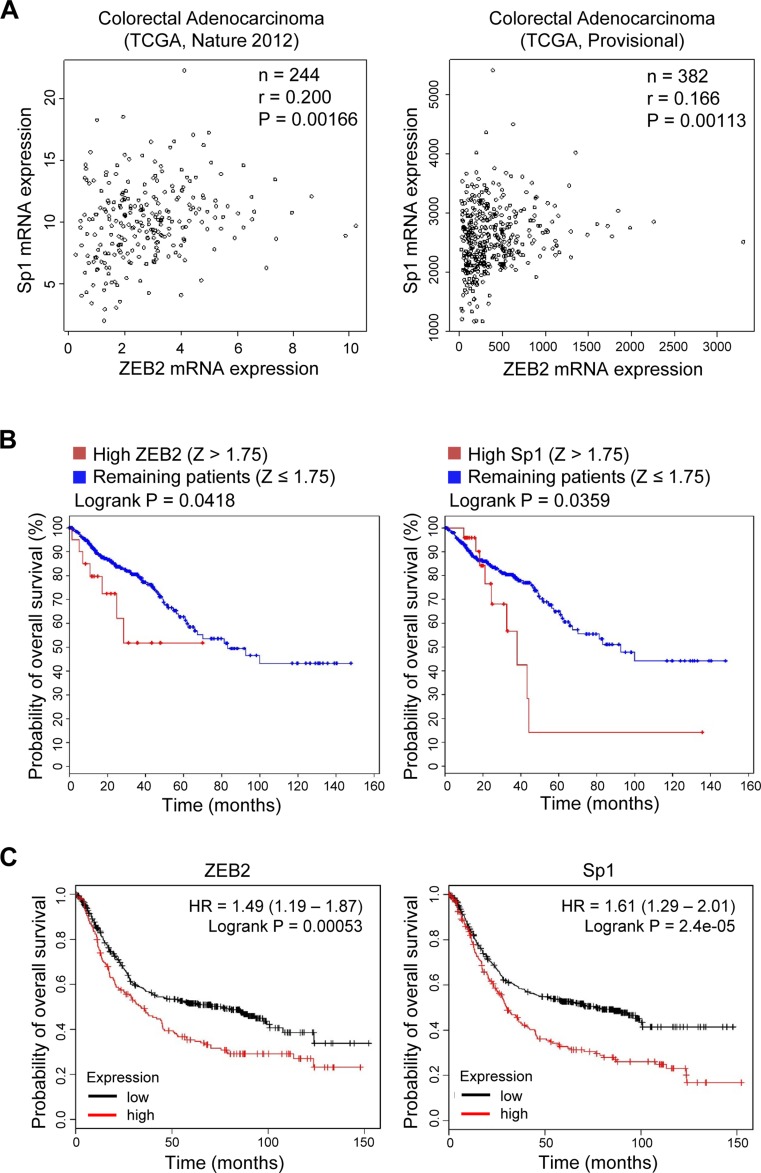
Positive association between ZEB2 and Sp1 expression in cancer patients (**A**) Scatter plots examining ZEB2 mRNA expression (x-axis) and Sp1 mRNA expression (y-axis) from colorectal adenocarcinoma data (left, TCGA, Nature 2012; right, TCGA, Provisional). Statistical analysis to assess the correlation was performed by Pearson’s test. (**B**) Kaplan–Meier analysis shows the probability of overall survival from colorectal adenocarcinoma patient data (TCGA, Provisional) in relation to ZEB2 and Sp1 mRNA expression. All tumors with an mRNA expression profile (*n* = 382) were analyzed. High ZEB2 expression was defined by *Z* > 1.75 and high Sp1 expression was defined by *Z* > 1.75. (**C**) Kaplan–Meier analysis shows the probability of overall survival from gastric cancer patient data (kmPlotter; *n* = 631) in relation to ZEB2 and Sp1 mRNA expression. ZEB2 and Sp1 expression was stratified as high vs. low according to the auto select best cutoff, and survival plots within previously published data sets were generated using http://kmplot.com (probe: 228333_at and 1553685_s_at for ZEB2 and Sp1, respectively) [43]. *P* values were calculated by the Logrank test.

To determine whether ZEB2 expression correlates with Sp1 expression in human cancers, we analyzed TCGA-generated colorectal adenocarcinoma data (two cancer studies; TCGA, Nature 2012 [20], and TCGA, Provisional). The correlation was analyzed by calculating Pearson’s correlation coefficient (r). ZEB2 expression was significantly correlated with Sp1 expression in human colorectal cancers (*n =* 244, *r* = 0.200, *P* = 0.00166 and *n =* 382, *r* = 0.166, *P* = 0.00113 for TCGA, Nature 2012, and TCGA, Provisional, studies, respectively) (Figure 7A).

In addition, colorectal cancer patients (TCGA, Provisional) whose tumors expressed higher levels of ZEB2 (*Z >* 1.75) or Sp1 (*Z >* 1.75) had a significantly worse overall survival than patients with lower levels of ZEB2 (*Z* ≤ 1.75) or Sp1 (*Z* ≤ 1.75) in their tumors (*n =* 382, *P* = 0.0418 and *P* = 0.0359 for ZEB2 and Sp1, respectively) (Figure 7B). Gastric cancer patients whose tumors highly expressed ZEB2 or Sp1 had a lower overall survival than those whose tumors did not (*n =* 631, *P* = 0.00053 and *P* = 0.000024 for ZEB2 and Sp1, respectively) when survival within previously published data sets was analyzed using kmPlotter (Figure 7C).

## DISCUSSION

We previously observed that ZEB2 upregulates expression of mesenchymal genes such as vimentin, integrin α5, and cadherin-11 to induce invasion and EMT [12, 13]. The present study demonstrates that ZEB2 induces expression of Sp1-regulated genes such as survivin, bcl-2, cyclin D1, and VEGF by cooperating with Sp1 to promote cancer cell survival and endothelial cell activation directly during metastasis. Our study suggests that cancer cells accelerate the aggressiveness of a malignancy by themselves via cooperation between ZEB2 and Sp1 and subsequent acquirement of a proliferative and viable phenotype. At the same time, ZEB2 induces endothelial cell activation and angiogenesis through a paracrine mechanism. ZEB2-mediated tumor cell survival and angiogenesis leading to distant organ colonization, may represent a novel pathway via which ZEB2 contributes to distant metastasis.

Recent studies reported that tumor cells may undergo MET, the reverse process of EMT, to colonize distant organs [8, 22], which is induced by downregulation of the EMT-inducing transcription factors Twist1 [23] and Prrx1 [24]. Clinically, Prrx1 expression (in primary tumors) is associated with good prognosis and metastasis-free disease, supporting the idea that reversal of EMT by loss of Prrx1 is required for macrometastasis [24]. By contrast, expression of other well-known EMT-inducing transcription factors such as Twist1, Snail1/2, and ZEB1/2 is usually associated with poor prognosis and metastasis in various cancer types [6], which is inconsistent with the concept that the reversal of EMT through reduction of EMT-inducing transcription factors is necessary for efficient metastatic colonization. In this study, analysis of TCGA and KmPlotter data also showed that high expression of ZEB2 or Sp1 is correlated with poor survival of colorectal and gastric cancer patients. Our study suggests that ZEB2 can cooperate with another transcription factor(s) to induce CTC survival and thus metastasis. This represents a novel pathway that could possibly explain how high expression of certain EMT-inducing transcription factors is associated with poor patient survival, although it needs to be determined whether and how disseminated cancer cells exhibit/acquire a proliferative phenotype in the presence of ZEB2. The ZEB2 level in the primary tumor vs. metastatic site also needs to be precisely investigated. Further studies of the functions and mechanisms of ZEB2 depending on the tumor progression stage are warranted.

It is intriguing that ZEB2 positively links cancer cell invasion with cell cycle progression and cell survival, given that cell proliferation and migration are mutually exclusive processes [25]. Activation of the cell cycle with concomitant inactivation of cell migration may be regarded as a genetically inherited compensatory mechanism to maintain cell homeostasis. Ectopic expression of Snail in Madin-Darby Canine Kidney cells induces decreased proliferation and accumulation of cells in G1 phase through direct repression of cyclin D1 and cyclin D2 transcription and induction of p21(Cip1) [26]. In particular, ZEB2 overexpression in A431 cells results in G1 cell cycle arrest through direct transcriptional repression of cyclin D1 [27], whereas ZEB2 appears to induce proliferation of hippocampal precursors *in vivo* [28]. Therefore, it is possible that ZEB2 can directly repress cyclin D1 transcription or induce cyclin D1 transcription through Sp1, depending on the cell type or context, although this remains to be explored.

Recent studies in human cancer patients and mouse tumor models report the presence of EMT markers in CTCs [21]. In a K-ras-driven mouse pancreatic tumor model, CTCs have a mesenchymal phenotype and express ZEB2, indicating activation of the EMT program in these cells [29]. Twist1 induction in a squamous cell carcinoma mouse tumor model dramatically increases the number of CTCs compared with control mice, and these CTCs have an EMT phenotype, indicating that activation of EMT directly promotes the production of CTCs [23]. In addition, tissue factor induced by EMT supports the persistence and survival of CTCs in the lungs of mice [30]. However, it largely remains unclear how EMT transcription factors contribute to CTC activation/persistence at the molecular level. Our study demonstrates that ZEB2 induces survival and subsequent seeding of CTCs, maybe through direct upregulation of pro-survival factors such as survivin and bcl-2, and thus probably contributes to cancer cell dissemination *in vivo*.

Most EMT-inducing transcription factors promote tumor angiogenesis *in vivo*, although the molecular mechanisms remain largely unclear. Expression of Twist1 is associated with enhanced tumor microvessel vasculature and VEGF expression in hepatocellular carcinomas [31]. ZEB1 is reported to upregulate VEGF expression and stimulate angiogenesis in breast cancer [32]. However, it has also been reported that ZEB1 can function as a negative regulator of angiogenesis *in vivo* [33]. Overexpression of Snail is associated with higher levels of the proangiogenic factors CXCL5 and CXCL8 [34], although the underlying mechanism is unclear. Our study suggests a direct association between ZEB2 expression and tumor angiogenesis; the ZEB2-Sp1 collaboration directly induces VEGF expression to stimulate endothelial cell activation and tumor angiogenesis.

Our previous reports [12, 13] and this study show that ZEB2 functions as a transcriptional activator by interacting with Sp1 to induce Sp1-regulated genes and thereby contributes not only to EMT/invasion but also to CTC survival and tumor angiogenesis. It is intriguing that ZEB2 enhanced Sp1 protein stability and Sp1 increased ZEB2 protein stability, which suggests the existence of a positive feedback loop between ZEB2 and Sp1. Clinical analysis provides evidence for cooperation between ZEB2 and Sp1. It remains unknown under which circumstances cooperation between ZEB2 and Sp1 occurs. It will be worth exploring the signal and mechanism responsible for cooperation between ZEB2 and Sp1.

Previously, we observed that nuclear expression of ZEB2 is higher in tumor cells than in normal cells, suggesting nuclear localization/transport of ZEB2 during tumor progression [13]. Along with ZEB1, ZEB2 is reported to localize to the cytoplasm as well as to the nucleus, and the subcellular localization of ZEB2 is regulated in normal and tumor tissues, suggesting that ZEB2 functions are regulated by means of its subcellular localization [35, 36]. It is possible that Sp1 modulates/enhances the nuclear presence of ZEB2, which can contribute to the accelerated aggressiveness of malignancy and tumor progression.

Sp-regulated genes are associated with cell proliferation (cyclin D1), metabolism (fatty acid synthase), apoptosis (bcl-2 and survivin), and angiogenesis (VEGF and VEGFR1) [16]. Metformin downregulates Sp transcription factors and expression of several Sp-regulated genes, resulting in anti-cancer activity [37]. Terameprocol, a semisynthetic derivative of a naturally occurring plant lignin, downregulates Sp1-mediated transcription of survivin to promote cancer cell apoptosis [38]. Therefore, these Sp1 inhibitors may have a therapeutic potential for the treatment of ZEB2-dependent metastatic cancers. The association between ZEB2 and Sp1 could also be targeted to develop novel therapies against cancer.

In conclusion, we demonstrate that, besides its role in cancer cell invasion, ZEB2 directly promotes CTC survival and tumor angiogenesis through cooperation with Sp1. Our findings suggest the presence of a positive feedback loop between ZEB2 and Sp1. This study contributes to our understanding of the diverse cellular functions of ZEB2 during tumor progression and their underlying mechanisms.

## MATERIALS AND METHODS

### Cell lines

Human embryonic kidney 293E (HEK293E) cells (American Type Culture Collection (ATCC), Manassas, VA, USA) were maintained in DMEM containing 10% fetal bovine serum (FBS) at 37°C in 5% CO_2_. SW480 (colon cancer; ATCC), SNU-398 (liver cancer), and SNU-638 (gastric cancer; Korean Cell Line Bank, Seoul, Korea) cells were maintained in RPMI1640 containing 10% FBS. HepG2 (liver cancer; ATCC) cells were maintained in Eagle’s Minimum Essential Medium containing 10% FBS. Human umbilical vein endothelial cells (HUVECs) were maintained using an EGM-2 BulletKit (Cambrex BioScience, Walkersville, MD, USA).

### Transfection of expression vectors and siRNA

Cells were transfected with a vector expressing ZEB2 (pCS3 SIP1; a kind gift from Dr. D. Huylebroeck, University of Leuven, Belgium) using Lipofectamine 2000 or electroporation (Invitrogen, Carlsbad, CA, USA). Cells were transfected with siRNA specific to ZEB2 (5′-CAACAUAUCCACUCCAUUU-3′), ZEB1 (5′-CUGUAAGAGAGAAGCGGAA-3′ for siZEB1#1 and 5′-GGUAGAUGGUAAUGUAAUA-3′ for siZEB1#2), or Sp1 (5′-GGUAGCUCUAAGUUUUGAU-3′) for 48 h using Lipofectamine 2000. The mutant ZEB2 lacking the Smad-binding domain (residues 437–487) (ZEB2∆SBD51) generated from pCS3 SIP1 was described previously [13].

### Immunoblot analysis

Whole-cell lysates were prepared using RIPA buffer as described previously [39] and analyzed using the following primary antibodies: anti-β-actin, anti-Sp1 (PEP2), anti-cyclin E, anti-ZEB1, and anti-GAPDH (Santa Cruz Biotechnology, Santa Cruz, CA, USA); anti-myc (Upstate Biotechnology, Lake Placid, NY, USA); anti-integrin α5 and anti-Tie2 (BD Biosciences, San Jose, CA, USA); anti-vimentin (Sigma, St Louis, MO, USA); anti-phospho-c-Jun N-terminal kinase (JNK) (T183/Y185), anti-phospho-extracellular signal-regulated kinase 1/2 (ERK1/2), anti-ERK1/2, anti-phospho-Akt (S473), anti-Akt, anti-survivin, anti-cyclin D1, anti-JNK, anti-phospho-VEGFR2 (Y996), anti-phospho-VEGFR2 (Y1175), anti-VEGFR2, anti-bcl-2, and anti-PARP (Cell Signaling, Danvers, MA, USA); anti-ZEB2 (6E5; Active Motif, Tokyo, Japan); anti-cyclin A, anti-bcl-2, anti-VEGF, and anti-phospho-c-Jun (Abcam, Cambridge, MA, USA); anti-ZO-3 (Invitrogen); and anti-E-cadherin and anti-phospho-Tie2 (R&D Systems, Minneapolis, MN, USA). Subcellular fractions were prepared using the Compartmental Protein Extraction Kit (Millipore, Billerica, MA, USA) according to the manufacturer’s instructions.

### Promoter reporter assay

Cells were transfected with Lipofectamine 2000. For transfection, 2 × 10^5^ cells were seeded onto 6-well plates. After incubation for 24 h, 2 µg of reporter plasmid DNA and 1.8 µg of the ZEB2 expression vector were co-transfected. At 48 h post-transfection, firefly luciferase activity was measured with a Dual-luciferase reporter assay system (Promega, Southampton, UK). The transfection efficiency was normalized by measuring Renilla luciferase activity encoded by the co-transfected Renilla luciferase vector (pRL-TK) or by measuring the fluorescence intensity of the co-transfected fluorescent dye-conjugated oligomer. The VEGF promoter (−2361/+298) construct was kindly provided by Dr. S.G. Chi (Korea University, Korea). The VEGF promoter (−267/+50) construct was kindly provided by Dr. G. Finkenzeller (Institute of Molecular Medicine, Germany). The mutant (Sp1- or Egr-1-binding sites) VEGF promoter reporter constructs generated from the construct containing the −85/+50 region were also provided by Dr. G. Finkenzeller.

### Analysis of secreted VEGF by an enzyme-linked immunosorbent assay (ELISA)

The amount of VEGF protein in the conditioned medium from cells was determined using the Human VEGF Quantikine ELISA kit (R&D Systems) according to the manufacturer’s instructions.

### Cell proliferation and 5-bromo-2′-deoxyuridine (BrdU) incorporation assays

Cell proliferation was determined by the colorimetric WST assay (Takara Bio Inc., Otsu, Shiga, Japan). Briefly, cells transfected with siRNA for 48 h were seeded into 96-well plates at a density of 4 × 10^3^ cells/well and incubated for 48 or 72 h in the presence of serum. Cells were then incubated with WST reagent (one-tenth of the medium volume), and formazan dye formation was determined by measuring absorbance at 450 nm using a spectrophotometric microplate reader (BMG LABTECH GmbH, Ortenber, Germany).

HUVECs (5 × 10^3^ cells/well) were seeded into 96-well plates, incubated for 24 h, and then further incubated for 24 h in the absence of serum. The cells were then incubated in the presence or absence of 10 µg/ml VEGF-blocking antibody (Avastin^®^; Roche, Basel, Switzerland) along with conditioned medium or 10 ng/ml VEGF for 48 h prior to the colorimetric WST assay.

BrdU incorporation analysis to measure DNA synthesis was performed using a Cell proliferation ELISA, BrdU (colorimetric) kit (Roche, Manheim, Germany) according to the manufacturer’s instructions. Briefly, cells transfected for 48 h were seeded into 96-well plates and incubated for 48 h. Then, the cells were incubated with 10 mM BrdU for 2 h before fixation and DNA denaturation. Cells were incubated with a peroxidase-conjugated antibody against BrdU. Color was developed with tetramethyl-benzidine substrate and analyzed by measuring absorbance at 370 nm (reference wavelength: 492 nm).

### Anchorage-independent soft agar assay

Cells were seeded at a density of 1 × 10^3^ cells/well in 6-well tissue culture plates in 0.3% agar (Sigma) over a 0.6% agar feed layer. Cells were allowed to grow at 37°C in 5% CO_2_ for 14 days, and the number of resulting colonies was counted per well.

### Cell survival analysis and anoikis assay

Cell survival under suspension culture conditions was determined. Briefly, cells were seeded into 96-well plates with an Ultra-Low Attachment Surface (Corning, NY, USA) at a density of 3 × 10^4^ cells/well and incubated for 3 or 5 days in the absence of serum. Cell viability was determined using the colorimetric WST assay as described above.

Cells (3 × 10^5^) were seeded into 6-well plates with an Ultra-Low Attachment Surface for 48 h to induce anoikis. Cells were washed and stained with annexin V and propidium iodide (PI) for 15 min at room temperature in the dark. The percentage of apoptotic cells was analyzed using flow cytometry.

### Invasion assay

Invasion assays were performed as described previously [12]. Cells were plated in serum-free medium on Transwell inserts (Corning) coated with 25 µg of Matrigel. The underside of the insert was pre-coated with 2 µg of collagen type I (Sigma). After incubation for 48 h at 37°C in 5% CO_2_, inserts were fixed with 3.7% paraformaldehyde prepared in phosphate-buffered saline (PBS) and stained with 2% crystal violet. The number of cells that had invaded was counted in five representative (×100) fields per insert.

### Reverse transcription-polymerase chain reaction (PCR)

Total RNA was isolated using TRIzol (Invitrogen), and cDNA was synthesized using reverse transcriptase (Bioneer, Daejon, Korea). Real-time quantitative PCR (qPCR) was performed using SYBR Green (PKT, Seoul, Korea) on a Rotor-Gene 6000 real-time rotary analyzer (Corbett, San Francisco, CA, USA) with ZEB2-specific primers (5′-TTGAGGAGACTGCCCAATAA-3′ and 5′-TATATCCAGGGCCCTACAGC-3′), survivin-specific primers (5′-ACTTGGCCCAGTGTTTCTTC-3′ and 5′-TCTTGACAGAAAGGAAAGCG-3′), cyclin D1-specific primers (5′-CTGTGCATCTACACCGACAA-3′ and 5′-CTTGAGCTTGTTCACCAGGA-3′), VEGF-specific primers (5′-AAATGCTTTCTCCGCTCTGA-3′ and 5′-CCCACTGAGGAGTCCAACAT-3′), and GAPDH-specific primers (5′-CATGACCACAGTCCATGCCAT-3′ and 5′-AAGGCCATGCCAGTGAGCTTC-3′) with an annealing temperature of 61°C.

### Generation of stable cell lines

ZEB2-specific siRNA (5′-CAACAUAUCCACUCCAUUU-3′) and scrambled siRNA (5′-AUUCUAUCCAAUACCUACC-3′) were subcloned into the pLKO.1 lentiviral shRNA vector (Addgene, Cambridge, MA, USA) to generate pLKO.1-shZEB2 and pLKO.1-shscrambled according to the manufacturer’s instructions. To generate lentiviruses, pLKO.1-shZEB2 or pLKO.1-shscrambled was co-transfected with Lentiviral Packaging Mix (Sigma) into Lenti-X-293T cells (Clontech) using Lipofectamine 2000, and virus-containing supernatants were harvested and concentrated at 48 h post-transfection. SNU-398 cells were transduced with the lentiviruses for 12 h in the presence of polybrene (4 µg/ml) and were subsequently selected with puromycin (0.7 µg/ml) for 2 weeks to establish stable clones.

### Mouse xenograft model

All animal procedures were performed in accordance with the guidelines of the Animal Care Committee at the Korea Research Institute of Bioscience and Biotechnology. Nude mice (BALB/c-nude, 5-week-old females) were obtained from Nara Biotech (Seoul, Korea). SNU-398 stable cells (ZEB2-suppressed cells and control cells) were injected subcutaneously into the right flank of each mouse (1 × 10^7^ cells per mouse; *n =* 8 per group). Body weight and tumor volume were measured twice per week. On day 26, mice were sacrificed and dissected tumor masses were fixed in 10% formalin and frozen in optimal cutting temperature compound (Sakura, Tokyo, Japan). The tumor volumes were calculated as follows: tumor volume = (a × b^2^) × 1/2, where a was the width at the widest point of the tumor and b was the maximal width perpendicular to a.

### Immunofluorescence staining

Optimal cutting temperature compound-embedded 6 µm-thick frozen tumor sections were processed for immunofluorescence analysis as per the standard protocol. Sections were blocked with 2% bovine serum albumin prepared in PBS and incubated with an anti-CD31 antibody (1:100 dilution; Santa Cruz Biotechnology) overnight at 4°C, followed by an Alexa546-conjugated goat anti-mouse secondary antibody (1:200 dilution; Thermo Fisher Scientific, Inc., Waltham, MA, USA). Sections were counterstained with 4,6-diamidino-2-phenylindole (DAPI; Sigma) to visualize cell nuclei. Immunofluorescence images were acquired under an Olympus DP30BW digital camera. CD31-positive cells present in tumor sections were quantified using MetaMorph software, version 7.1.6.0 (Molecular Devices, Sunnyvale, CA, USA).

HEK293E cells were plated on serum-coated coverslips and transfected with Sp1-specific siRNA and the ZEB2 expression vector for 48 h. Cells were fixed for 5 min in 10% formalin and permeabilized in 0.3% Triton X-100 for 3 min. Cells were incubated with an anti-ZEB2 antibody (Active Motif) followed by a Alexa546-conjugated secondary antibody and then with an anti-Sp1 antibody (Santa Cruz Biotechnology) followed by a FITC-conjugated secondary antibody. Cells were counterstained with DAPI. Mounted samples were visualized with a confocal microscope (LSM 510 META; Carl Zeiss, Jena, Germany).

### TUNEL staining

TUNEL staining was performed to measure apoptosis in optimal cutting temperature compound-embedded tumor sections using the ApopTag Plus Peroxidase *In Situ* Apoptosis Kit (Merck Millipore, Billerica, MA, USA) according to the manufacturer’s instructions. Cell nuclei were stained with hematoxylin.

### CTC survival analysis (early metastasis model)

SNU-398 stable cells (ZEB2-suppressed cells and control cells) were injected via the tail vein into 7-week-old nude mice (5 × 10^6^ cells per mouse; *n =* 4 per group). Mice were sacrificed 24 h after injection as reported [30]. Lungs were harvested, minced, and digested with proteinase K and RNase A at 56°C for 15 min. Total DNA was isolated from the lungs using the G-spin Total DNA Extraction Kit (Intron, Daejon, Korea). DNA concentrations were measured on a NanoDrop ND-1000 spectrophotometer (Thermo Fisher Scientific, Inc.).

To quantify survival and early seeding (arrest) of CTCs, human tumor cell contents present in mouse lungs were determined by modification of a previously reported method [40]. Briefly, real-time qPCR analysis of human prostaglandin E receptor 2 (PTGER2) genomic DNA was performed with a PTGER2-specific primer pair (5′-TACCTGCAGCTGTACGCCAC-3′ and 5′-GCCAGGAGAATGAGGTGGTC-3′) and a human PTGER2-specific probe (FAM 5′-TGCTGCTTCTCATTGTCTCG-3′ TAMRA) using QuantiTect Probe PCR Master Mix (Qiagen, Hilden, Germany) on a Rotor gene Q instrument (Qiagen) according to the manufacturer’s instructions. PCR was performed in triplicate with a final volume of 50 µl per reaction using 1 µg of total genomic DNA as a template. After denaturation for 15 min at 95°C, the reaction was continued for 40 cycles of 94°C for 15 sec and 60°C for 60 sec. The Threshold Cycle (*Ct*) values for each set of three reactions were averaged for all subsequent calculations.

In parallel, a standard curve was generated using genomic DNA extracted from SNU-398 cells and nude mouse lungs. Standard curve samples included serial dilutions of mouse-only, human-only, or human plus mouse mixed samples of known DNA concentrations. A standard curve with the equation of the linear trend line was developed by plotting the mean *Ct* values on the y-axis versus the log amount of human genomic DNA on the x-axis.

### Analysis of the cancer genome atlas (TCGA) and kmPlotter data

cBioPortal (www.cbioportal.org) [41, 42] was used to analyze TCGA-generated human colorectal adenocarcinoma data (two cancer studies; TCGA, Nature 2012 [20], and TCGA, Provisional). All patient samples where the mRNA expression profiles were available were included in our analysis per cancer study. Pearson’s correlation coefficient (r) and the *P*-value were calculated using the cbioportal webpage and CGDS-R package (available at http://cran.r-project.org/web/packages/cgdsr/index.html) tools. Survival curve analysis was performed using the cBioPortal webpage tools. Survival of gastric cancer patients within previously published data sets was analyzed using kmPlotter (http://kmplot.com) [43].

### Statistical analysis

Statistical analyses were performed using the Student’s *t*-test, Logrank test, and Pearson’s test. *P* < 0.05 was considered statistically significant.

## SUPPLEMENTARY MATERIALS FIGURES



## Abbreviations

BrdU: 5-bromo-2′-deoxyuridine
CTC: circulating tumor cell
ELISA: enzyme-linked immunosorbent assay
EMT: epithelial-mesenchymal transition
ERK1/2: extracellular signal-regulated kinase 1/2
HEK293E: human embryonic kidney 293E
HUVEC: human umbilical vein endothelial cell
JNK: c-Jun N-terminal kinase
MVD: microvessel density
PTGER2: prostaglandin E receptor 2
qPCR: quantitative polymerase chain reaction
siRNA: small interfering RNA
TCGA: The Cancer Genome Atlas
VEGF: vascular endothelial growth factor

## References

[1] Chaffer CL, Weinberg RA (2011). A perspective on cancer cell metastasis. Science.

[2] Yang J, Weinberg RA (2008). Epithelial-mesenchymal transition: at the crossroads of development and tumor metastasis. Dev Cell.

[3] Thiery JP, Acloque H, Huang RY, Nieto MA (2009). Epithelial-mesenchymal transitions in development and disease. Cell.

[4] Thiery JP, Sleeman JP (2006). Complex networks orchestrate epithelial-mesenchymal transitions. Nat Rev Mol Cell Biol.

[5] Zeisberg M, Neilson EG (2009). Biomarkers for epithelial-mesenchymal transitions. J Clin Invest.

[6] Peinado H, Olmeda D, Cano A (2007). Snail, Zeb and bHLH factors in tumour progression: an alliance against the epithelial phenotype?. Nat Rev Cancer.

[7] Sanchez-Tillo E, Liu Y, de Barrios O, Siles L, Fanlo L, Cuatrecasas M, Darling DS, Dean DC, Castells A, Postigo A (2012). EMT-activating transcription factors in cancer: beyond EMT and tumor invasiveness. Cell Mol Life Sci.

[8] Zheng H, Kang Y (2014). Multilayer control of the EMT master regulators. Oncogene.

[9] Chua HL, Bhat-Nakshatri P, Clare SE, Morimiya A, Badve S, Nakshatri H (2007). NF-kappaB represses E-cadherin expression and enhances epithelial to mesenchymal transition of mammary epithelial cells: potential involvement of ZEB-1 and ZEB-2. Oncogene.

[10] Sayan AE, Griffiths TR, Pal R, Browne GJ, Ruddick A, Yagci T, Edwards R, Mayer NJ, Qazi H, Goyal S, Fernandez S, Straatman K, Jones GD (2009). SIP1 protein protects cells from DNA damage-induced apoptosis and has independent prognostic value in bladder cancer. Proc Natl Acad Sci USA.

[11] Browne G, Sayan AE, Tulchinsky E (2010). ZEB proteins link cell motility with cell cycle control and cell survival in cancer. Cell Cycle.

[12] Nam EH, Lee Y, Park YK, Lee JW, Kim S (2012). ZEB2 upregulates integrin alpha5 expression through cooperation with Sp1 to induce invasion during epithelial-mesenchymal transition of human cancer cells. Carcinogenesis.

[13] Nam EH, Lee Y, Zhao XF, Park YK, Lee JW, Kim S (2014). ZEB2-Sp1 cooperation induces invasion by upregulating cadherin-11 and integrin alpha5 expression. Carcinogenesis.

[14] Black AR, Black JD, Azizkhan-Clifford J (2001). Sp1 and kruppel-like factor family of transcription factors in cell growth regulation and cancer. J Cell Physiol.

[15] Li L, Davie JR (2010). The role of Sp1 and Sp3 in normal and cancer cell biology. Ann Anat.

[16] Abdelrahim M, Baker CH, Abbruzzese JL, Safe S (2006). Tolfenamic acid and pancreatic cancer growth, angiogenesis, and Sp protein degradation. J Natl Cancer Inst.

[17] Finkenzeller G, Sparacio A, Technau A, Marme D, Siemeister G (1997). Sp1 recognition sites in the proximal promoter of the human vascular endothelial growth factor gene are essential for platelet-derived growth factor-induced gene expression. Oncogene.

[18] Shimoyamada H, Yazawa T, Sato H, Okudela K, Ishii J, Sakaeda M, Kashiwagi K, Suzuki T, Mitsui H, Woo T, Tajiri M, Ohmori T, Ogura T (2010). Early growth response-1 induces and enhances vascular endothelial growth factor-A expression in lung cancer cells. Am J Pathol.

[19] Koch S, Tugues S, Li X, Gualandi L, Claesson-Welsh L (2011). Signal transduction by vascular endothelial growth factor receptors. Biochem J.

[20] Cancer Genome Atlas Network (2012). Comprehensive molecular characterization of human colon and rectal cancer. Nature.

[21] Tsai JH, Yang J (2013). Epithelial-mesenchymal plasticity in carcinoma metastasis. Genes Dev.

[22] Brabletz T (2012). EMT and MET in metastasis: where are the cancer stem cells?. Cancer Cell.

[23] Tsai JH, Donaher JL, Murphy DA, Chau S, Yang J (2012). Spatiotemporal regulation of epithelial-mesenchymal transition is essential for squamous cell carcinoma metastasis. Cancer Cell.

[24] Ocana OH, Corcoles R, Fabra A, Moreno-Bueno G, Acloque H, Vega S, Barrallo-Gimeno A, Cano A, Nieto MA (2012). Metastatic colonization requires the repression of the epithelial-mesenchymal transition inducer Prrx1. Cancer Cell.

[25] Zheng PP, Severijnen LA, van der Weiden M, Willemsen R, Kros JM (2009). Cell proliferation and migration are mutually exclusive cellular phenomena *in vivo*: implications for cancer therapeutic strategies. Cell Cycle.

[26] Vega S, Morales AV, Ocana OH, Valdes F, Fabregat I, Nieto MA (2004). Snail blocks the cell cycle and confers resistance to cell death. Genes Dev.

[27] Mejlvang J, Kriajevska M, Vandewalle C, Chernova T, Sayan AE, Berx G, Mellon JK, Tulchinsky E (2007). Direct repression of cyclin D1 by SIP1 attenuates cell cycle progression in cells undergoing an epithelial mesenchymal transition. Mol Biol Cell.

[28] Miquelajauregui A, Van de Putte T, Polyakov A, Nityanandam A, Boppana S, Seuntjens E, Karabinos A, Higashi Y, Huylebroeck D, Tarabykin V (2007). Smad-interacting protein-1 (Zfhx1b) acts upstream of Wnt signaling in the mouse hippocampus and controls its formation. Proc Natl Acad Sci USA.

[29] Rhim AD, Mirek ET, Aiello NM, Maitra A, Bailey JM, McAllister F, Reichert M, Beatty GL, Rustgi AK, Vonderheide RH, Leach SD, Stanger BZ (2012). EMT and dissemination precede pancreatic tumor formation. Cell.

[30] Bourcy M, Suarez-Carmona M, Lambert J, Francart ME, Schroeder H, Delierneux C, Skrypek N, Thompson EW, Jerusalem G, Berx G, Thiry M, Blacher S, Hollier BG (2016). Tissue Factor Induced by Epithelial-Mesenchymal Transition Triggers a Procoagulant State That Drives Metastasis of Circulating Tumor Cells. Cancer Res.

[31] Niu RF, Zhang L, Xi GM, Wei XY, Yang Y, Shi YR, Hao XS (2007). Up-regulation of Twist induces angiogenesis and correlates with metastasis in hepatocellular carcinoma. J Exp Clin Cancer Res.

[32] Liu L, Tong Q, Liu S, Cui J, Zhang Q, Sun W, Yang S (2016). ZEB1 Upregulates VEGF Expression and Stimulates Angiogenesis in Breast Cancer. PLoS One.

[33] Inuzuka T, Tsuda M, Tanaka S, Kawaguchi H, Higashi Y, Ohba Y (2009). Integral role of transcription factor 8 in the negative regulation of tumor angiogenesis. Cancer Res.

[34] Yanagawa J, Walser TC, Zhu LX, Hong L, Fishbein MC, Mah V, Chia D, Goodglick L, Elashoff DA, Luo J, Magyar CE, Dohadwala M, Lee JM (2009). Snail promotes CXCR2 ligand-dependent tumor progression in non-small cell lung carcinoma. Clin Cancer Res.

[35] Oztas E, Avci ME, Ozcan A, Sayan AE, Tulchinsky E, Yagci T (2010). Novel monoclonal antibodies detect Smad-interacting protein 1 (SIP1) in the cytoplasm of human cells from multiple tumor tissue arrays. Exp Mol Pathol.

[36] Prislei S, Martinelli E, Zannoni GF, Petrillo M, Filippetti F, Mariani M, Mozzetti S, Raspaglio G, Scambia G, Ferlini C (2015). Role and prognostic significance of the epithelial-mesenchymal transition factor ZEB2 in ovarian cancer. Oncotarget.

[37] Nair V, Pathi S, Jutooru I, Sreevalsan S, Basha R, Abdelrahim M, Samudio I, Safe S (2013). Metformin inhibits pancreatic cancer cell and tumor growth and downregulates Sp transcription factors. Carcinogenesis.

[38] Chang CC, Heller JD, Kuo J, Huang RC (2004). Tetra-O-methyl nordihydroguaiaretic acid induces growth arrest and cellular apoptosis by inhibiting Cdc2 and survivin expression. Proc Natl Acad Sci USA.

[39] Lee Y, Ko D, Min HJ, Kim SB, Ahn HM, Lee Y, Kim S (2016). TMPRSS4 induces invasion and proliferation of prostate cancer cells through induction of Slug and cyclin D1. Oncotarget.

[40] Alcoser SY, Kimmel DJ, Borgel SD, Carter JP, Dougherty KM, Hollingshead MG (2011). Real-time PCR-based assay to quantify the relative amount of human and mouse tissue present in tumor xenografts. BMC Biotechnol.

[41] Gao J, Aksoy BA, Dogrusoz U, Dresdner G, Gross B, Sumer SO, Sun Y, Jacobsen A, Sinha R, Larsson E, Cerami E, Sander C, Schultz N (2013). Integrative analysis of complex cancer genomics and clinical profiles using the cBioPortal. Sci Signal.

[42] Cerami E, Gao J, Dogrusoz U, Gross BE, Sumer SO, Aksoy BA, Jacobsen A, Byrne CJ, Heuer ML, Larsson E, Antipin Y, Reva B, Goldberg AP (2012). The cBio cancer genomics portal: an open platform for exploring multidimensional cancer genomics data. Cancer Discov.

[43] Szász AM, Lánczky A, Nagy Á, Förster S, Hark K, Green JE, Boussioutas A, Busuttil R, Szabó A, Győrffy B (2016). Cross-validation of survival associated biomarkers in gastric cancer using transcriptomic data of 1,065 patients. Oncotarget.

