# Supplementary material for: Cooperation between ZEB2 and Sp1 promotes cancer cell survival and angiogenesis during metastasis through induction of survivin and VEGF
Source: Oncotarget. 2017 Dec 11;9(1):726–42. doi: 10.18632/oncotarget.23139 (PMC5787504; doi:10.18632/oncotarget.23139)
Supplement: Supplementary file 1 [file oncotarget-09-726-s001.pdf]

Cooperation between ZEB2 and Sp1 promotes cancer cell survival and angiogenesis during metastasis through induction of survivin and VEGF

SUPPLEMENTARY MATERIALS

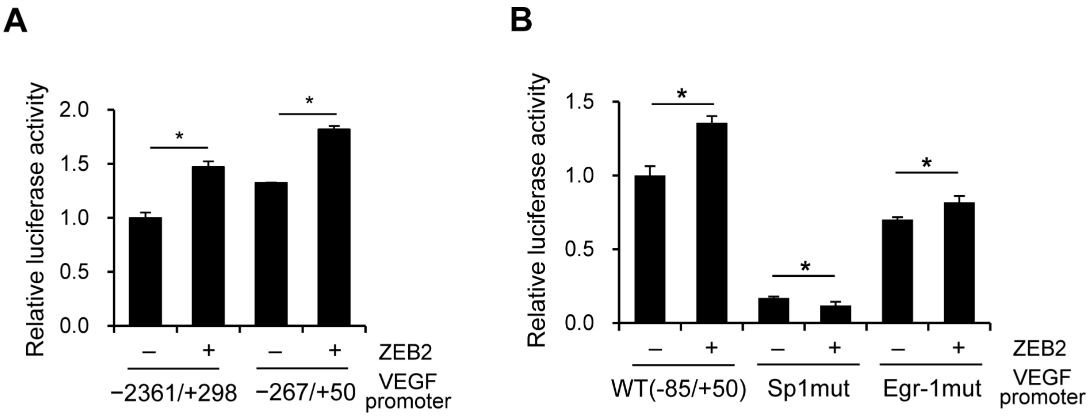

Supplementary Figure 1: (**A**) HEK293E cells were co-transfected with a ZEB2 expression vector and VEGF promoter (−2361/+298 and −267/+50) reporter constructs for 48 h. Firefly luciferase activity representing VEGF promoter activity was measured after 48 h and normalized to Renilla luciferase activity to determine the transfection efficiency. (**B**) Mutation analysis of Sp1 sites and Egr-1 sites in the VEGF promoter (−85/+50). Reporter constructs containing Sp1 site or Egr-1 site mutations were used in the reporter assay using HEK293E cells. Values represent mean ± standard deviation. $^{*}P < 0.05$.

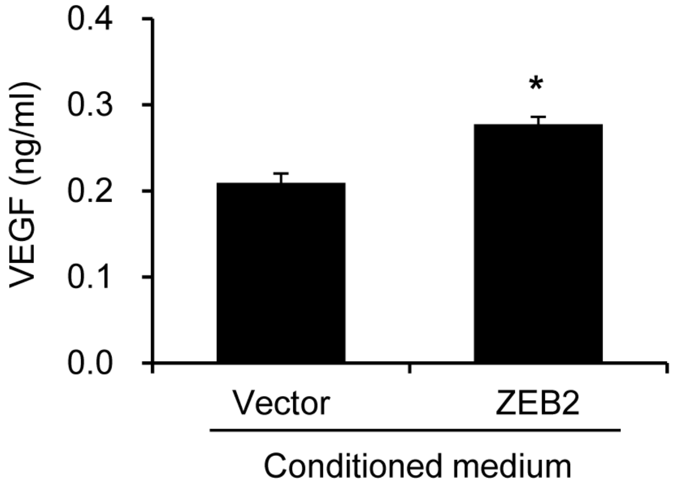

Supplementary Figure 2: HEK293E cells were transfected with a ZEB2 expression vector for 48 h. Conditioned medium from transfected cells was collected for an additional 48 h. VEGF levels in conditioned medium were determined by an ELISA.

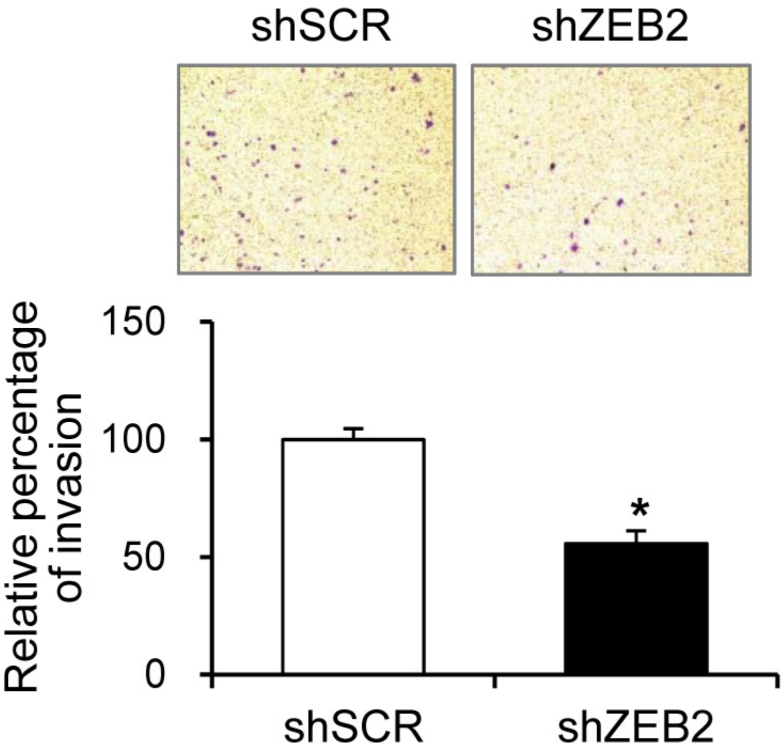

Supplementary Figure 3: ZEB2-suppressed SNU-398 stable cells were allowed to invade Matrigel (5×10^4 cells) for 48 h. The number of cells that had invaded was counted in five representative (×100) fields per Transwell insert. Values represent mean ± standard deviation. $^*P < 0.05$. shSCR, scrambled shRNA.

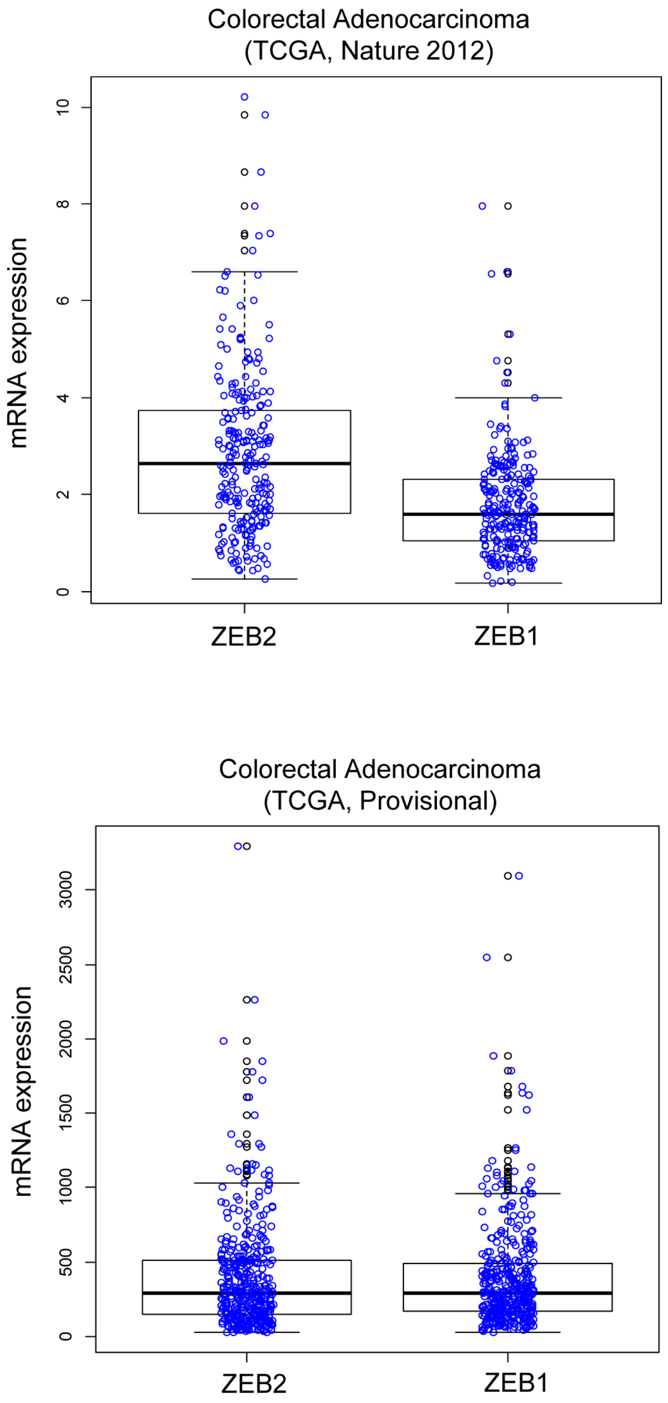

Supplementary Figure 4: Expression of ZEB2 and ZEB1 in human colorectal cancers. The mean (± standard deviation) levels of ZEB2 mRNA versus ZEB1 mRNA are 2.85 ± 1.7 versus 1.76 ± 1.07 (*n* = 244, *P* < 0.001; Upper panel) and 393.0 ± 371 versus 390.9 ± 356 (*n* = 382, *P* = 0.94; Lower panel). Of note, the mean (± standard deviation) levels of GAPDH mRNA were 3110 ± 1772 (Upper panel) and 78089 ± 32960 (Lower panel). Statistical analysis was performed using Student's *t*-test.

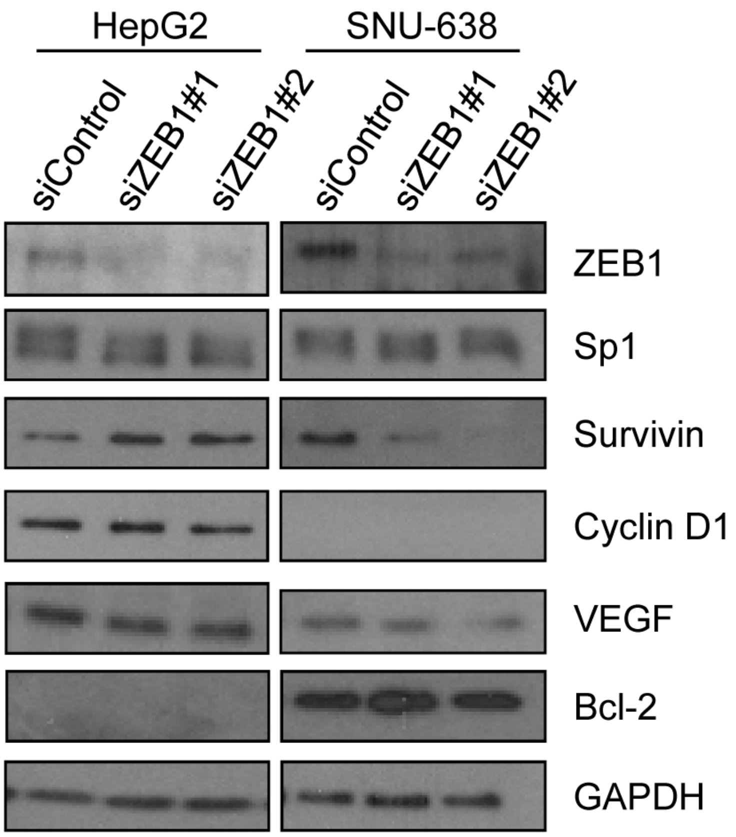

Supplementary Figure 5: HepG2 and SNU-638 cells were transfected with ZEB1-specific siRNAs for 48 h and 72 h, respectively. Transfected cells were then lysed for immunoblot analysis. Of note, bcl-2 and cyclin D1 were not detected in HepG2 and SNU-638 cells, respectively.

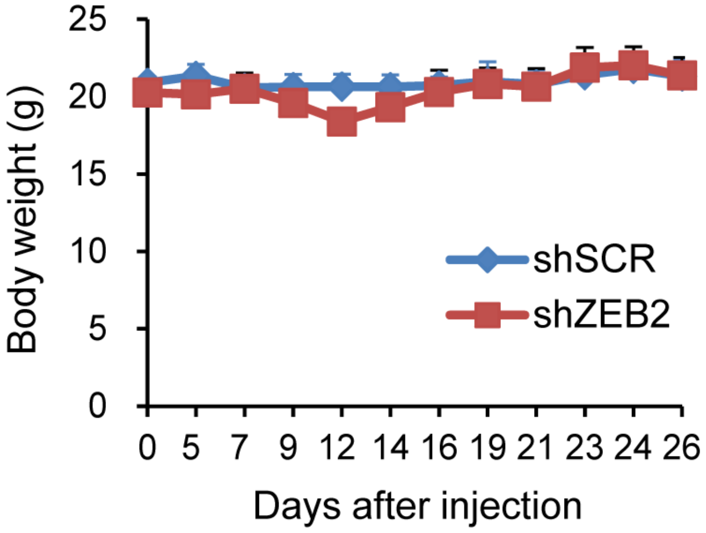

Supplementary Figure 6: SNU-398 stable cells (ZEB2-suppressed cells and control cells) were injected subcutaneously into the flanks of nude mice (*n* = 8; 1 × 10^7 cells/mouse) as described in the Materials and Methods. Body weight was then monitored for 26 days. Values represent mean ± standard deviation. shSCR, scrambled shRNA.

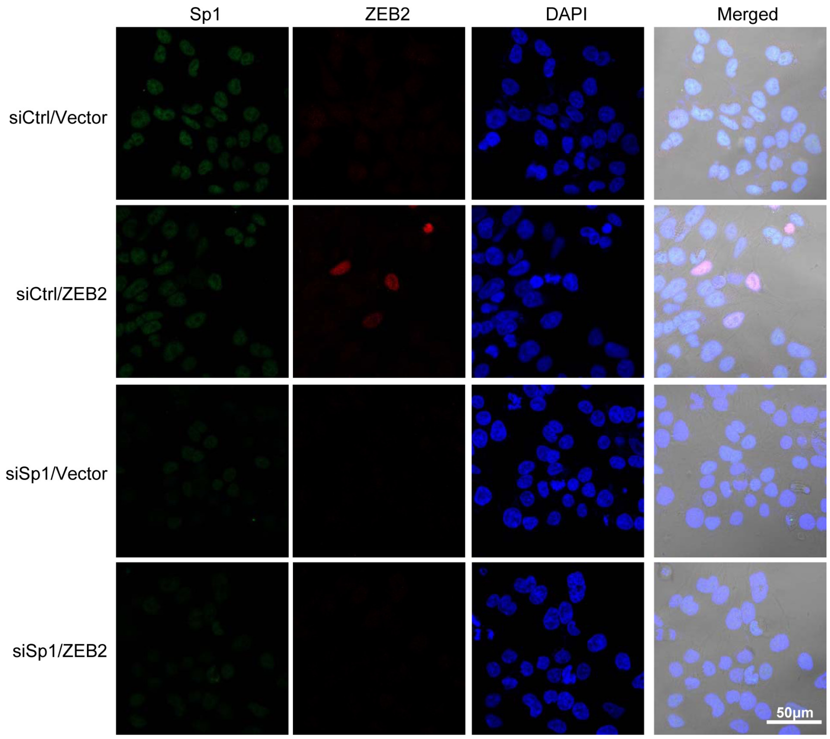

Supplementary Figure 7: HEK293E cells were transfected with a ZEB2 expression vector and siRNA specific to Sp1 for 48 h and then subjected to immunofluorescence analysis of ZEB2 (red), Sp1 (green), and nuclei (blue). siCtrl, control siRNA. Bar, 50 µm.
